# Argyrin B exhibits potent therapeutic efficacy in a *Clostridioides difficile*-infection mouse model while preserving microbiota functionality

**DOI:** 10.1038/s44259-026-00248-x

**Published:** 2026-07-16

**Authors:** Sari Rasheed, Katrin Ehrhardt, Ahmed Mohamed Mostafa Abdrabou, Alexander Mellmann, Anna Lechleiter, Domen Pogorevc, Sabryna Junker, Lutz von Müller, Marius Vital, Jennifer Herrmann, Guntram A. Grassl, Markus Bischoff, Rolf Müller

**Affiliations:** 1 Department of Microbial Natural Products, Helmholtz Institute for Pharmaceutical Research Saarland (HIPS)/Helmholtz Centre for Infection Research (HZI), Saarbrücken, Germany; 2 German Center for Infection Research (DZIF) partnersite, Hannover-Braunschweig, Germany; 3 PharmaScienceHub (PSH), Saarland University Campus, Saarbrücken, Germany; 4 Institute of Medical Microbiology and Hospital Epidemiology, Hannover Medical School, Hannover, Germany; 5 Institute for Medical Microbiology and Hygiene, Saarland University, Homburg/Saar, Germany; 6 Medical Microbiology and Immunology Department, Faculty of Medicine, Mansoura University, Mansoura, Egypt; 7 German National Reference Center for Clostridioides (Clostridium) difficile, Homburg/Saar–Münster–Coesfeld, Germany; 8 Institute of Hygiene, University of Münster, Münster, Germany; 9 Institute for Laboratory Medicine, Microbiology and Hygiene, Christophorus Kliniken, Coesfeld, Germany; 10 Cluster of Excellence RESIST (EXC 2155), Hannover Medical School, Hannover, Germany; 11 Department of Pharmacy, Saarland University, Saarbrücken, Germany

**Keywords:** Diseases, Drug discovery, Microbiology

## Abstract

*Clostridioides difficile* infection (CDI) remains a leading cause of antibiotic-associated diarrhoea, with high recurrence rates and limited treatment options that preserve gut microbiota. Current treatments, including vancomycin, further disrupt the already compromised gut microbiota, often prolonging dysbiosis and increasing the risk of colonization by resistant pathogens. This study demonstrates that argyrin B exhibits potent activity against *C. difficile* in vitro and significantly reduces bacterial burden in a mouse model of infection. Argyrin B displayed a narrow antimicrobial spectrum, suggesting that commensal gut bacteria are hardly affected, and may allow for a faster restoration of the antibiotic pre-damaged gut microbiota. The compound exhibited a pharmacokinetic profile characterized by low systemic absorption and elevated colonic concentrations, representing favourable characteristics. The compound acts through a novel mechanism by targeting elongation factor G, distinct from existing therapies, and resistance emerged at low frequency via point mutations in the target gene. These features suggest that argyrin B may offer a novel and well-tolerated therapeutic approach for CDI, with potential to support microbiota preservation, which may contribute to reduced recurrence risk.

## Introduction

*Clostridioides difficile* (*C. difficile*) is an anaerobic Gram-positive, spore-forming, and toxin-producing bacterium, commonly found in the environment as well as in human and animal intestinal tracts, and its spores are transmitted by the faecal–oral route^1^. Over the past decades, the incidence of *C. difficile* infection (CDI) has increased globally, and CDI has become one of the leading causes of healthcare-associated infections, connected to high morbidity and mortality^2,3^. *C. difficile* colonization has been reported in neonatal animals and humans, with higher colonization rates generally observed early in life. Colonization patterns appear to change with host maturation and dietary transition, which has been associated with the establishment of a more complex intestinal microbiota and the development of colonization resistance^4,5^. In healthy microbiota, colonization resistance against *C. difficile* is sustained through multiple mechanisms, including competition for nutrients and niches, production of antimicrobial compounds, and stimulation of the host immune response^6–9^.

Over the past decade, treatment recommendations for CDI have shifted away from metronidazole toward vancomycin or fidaxomicin as first-line therapy^10^. Fidaxomicin, a macrocyclic RNA polymerase (RNAP) inhibitor with a narrow antibacterial spectrum and minimal systemic absorption, has been shown in randomized trials and guideline reviews to reduce the risk of recurrent CDI (rCDI) compared with vancomycin^11–13^. Additional treatment options include bezlotoxumab, a human monoclonal antibody targeting *C. difficile* toxin B, and faecal microbiota transplantation (FMT) for the treatment of rCDI^14,15^. Despite advances in treatment, the persistent global burden of CDI, emerging reports of reduced susceptibility to fidaxomicin, together with the considerable risk of recurrence following antibiotic therapy, highlight the need for novel antibacterial agents^13,16–18^.

Antibiotic-associated diarrhoea (AAD) occurs due to an imbalance in gut microbiota resulting from antibiotic use^19,20^. While most instances of AAD are mild and self-limiting, in the presence of toxigenic *C. difficile* in the gut microbiota of the patient, a CDI may develop. Toxigenic *C. difficile* poses a significant public health threat and ranks among the most prevalent healthcare-associated infections worldwide^1,21^*. C. difficile* spreads via the faecal–oral route and toxigenic variants cause disease by producing two protein exotoxins, toxin A and toxin B, which are cytotoxic to colonic epithelial cells^19,20,22^. The primary objectives in the management of CDI are to achieve clinical cure and prevent recurrence. Although current guidelines offer clear recommendations for the treatment of primary CDI, recurrent CDI remains a significant therapeutic challenge, with limited effective options available. According to the World Health Organization’s 2025 report on antibacterial agents in clinical and preclinical development, 4 of the 50 traditional antibacterial agents currently in the clinical pipeline are being developed to treat *C. difficile* infections^23^. Among these, ibezapolstat, a first-in-class DNA polymerase IIIC inhibitor lacking cross-resistance with currently approved treatments for CDI (metronidazole, vancomycin, and fidaxomicin), and thus increases the number of innovative agents to three of the four traditional candidates (75%). In addition, 11 of the 40 non-traditional antibacterial agents in clinical development target *C. difficile* infections. One of these, the live biotherapeutic product VE303, designed to prevent rCDI through microbiota restoration, has advanced to Phase 3 clinical evaluation. Across clinical development stages, five non-traditional agents targeting *C. difficile* are in Phase 1 (out of 27 total Phase 1 compounds), while in Phase 1/2 and Phase 2, there are six agents (three traditional and three non-traditional) among 46 total compounds. In the most advanced stages (Phase 2/3 and Phase 3), four agents target *C. difficile*, including one traditional and three non-traditional products, out of 14 total late-stage compounds in the pipeline^23^.

Argyrins represent a family of cyclic octapeptides (Fig. S1), which are produced by the myxobacterial strains *Archangium gephyra* Ar8082 and *Cystobacter* sp. SBCb004. They exhibit a distinct antibacterial activity profile, demonstrating pronounced activity against *C. difficile*, *Mycoplasma gallisepticum*, *Neisseria caviae*, *Pseudomonas aeruginosa*, *Burkholderia multivorans* and *Stenotrophomonas maltophilia*^24–27^. Studies have identified the elongation factor G (EF-G) as the cellular target for argyrin in *P. aeruginosa*. Argyrins were shown to bind to EF-G at a unique allosteric pocket, causing EF-G to adopt an elongated conformation that is unsuitable for ribosome binding, consequently, hindering protein synthesis^28–30^. The same target is also addressed by the steroid anti-staphylococcal antibiotic fusidic acid (FA), however, mutagenesis studies and structural analysis have shown that argyrin binds to an alternative site of EF-G that clearly differs from that of (FA)^28–30^. Recent studies have shown that argyrin B does not inhibit the binding of EF-G to the ribosome; instead, it specifically binds to EF-G on the ribosome, capturing a late intermediate state of the translocation process through a binding pocket that is functionally connected yet physically separate from FA^31^. We previously established a heterologous production platform based on engineered *Myxococcus xanthus* to enhance argyrin biosynthesis^32^. Through optimization of transcription and translation initiation rates, along with increased gene dosage, we significantly improved total argyrin yields, reaching titre of approximately 400 mg/L. This advancement ensures a reliable compound supply and marks a key step toward the preclinical development of argyrins. In this study, we evaluated the in vitro activity of argyrin B against clinical *C. difficile* isolates and observed potent, selective antibacterial effects. Based on these findings, we proceeded to assess its pharmacokinetic properties and in vivo efficacy in a preclinical model of *C. difficile* infection, identifying argyrin B as a promising targeted therapeutic agent.

## Results

### In vitro activity testing against *C. difficile* and *Clostridium* spp

A library of 259 purified myxobacterial natural products as part of the DZIF (German Center for Infection Research) compound repository was screened against *C. difficile*^33^. Intriguingly, potent inhibition of *C. difficile* growth was observed for 17 compounds that were initially tested at 10 µM (Table S1). While some compound classes were deprioritized due to known activity against *C. difficile* (chlorotonil)^34^, prioritized development in other indications (corallopyronin)^35^, unspecific/toxic activity (tartrolons, tubulysin)^36,37^, or limited availability of low-titer derivatives (icumazol, leupyrrin, pentacaronic acid, soraphen, terrestribisamid), three natural product classes were selected for further studies. The RNA polymerase inhibitor sorangicin A^38,39^ and the lipid II binder katanosin B (also known as lysobactin)^40^ displayed minimum inhibitory concentrations (MICs) in the sub-µg/mL range against *C. difficile* strain DSM 27147 (ribotype 027) but also inhibited commensal *Bacteroides* sp. (*B. fragilis* DSM 2151). Argyrins were the most promising hits with nanomolar potency against *C. difficile* (MIC ≤ 0.03 µg/mL) and no activity against *B. fragilis* (>64 µg/mL) (Table S2). Prior studies have documented their antimicrobial activity primarily against Gram-negative pathogens such as *P. aeruginosa*, *S. maltophilia*, and *B. multivorans*. These compounds also show activity against membrane-compromised strains of *Escherichia coli* and *Salmonella enterica* serovar Typhimurium^27,41^.

To our knowledge, argyrin activity against *C. difficile* has only been briefly noted in a few reports, without detailed investigation. Our study presents the first systematic evaluation of argyrins against this clinically relevant pathogen, thereby expanding their known antimicrobial spectrum^25^. The in vitro activity of three representative derivatives, argyrins B, C, and D, was evaluated using broth microdilution assays against *C. difficile* strains DSM 28645 (ribotype [RT] 012) and DSM 27147 (RT 027). Argyrin B and argyrin C particularly demonstrated potent in vitro activity, exhibiting an MIC in the low ng/mL range against both *C. difficile* strains (MIC 18–37.5 and 25 ng/mL, respectively). In comparison, argyrin D showed an MIC of 75–100 ng/mL (Table 1A). Among the natural product derivatives, argyrin B can be produced at most reasonable titres from the natural host and at larger quantities from an engineered heterologous producer^32^. Thus, we focused our subsequent studies on the biological and pharmacological characterization of argyrin B. We assessed argyrin B against a panel of fifty-one (51) clinical *C. difficile* isolates to determine the MIC_50_ and MIC_90_ values. The assembled panel of clinical isolates represented a wide range of RTs, including hypervirulent RTs such as RT027, RT078 and RT176. The panel also reflected the complex antimicrobial resistance characteristics of *C. difficile* for antibiotics considered to increase the risk of CDI, such as the fluoroquinolone moxifloxacin or the macrolide antibiotic clarithromycin. However, all isolates in the panel remained susceptible to vancomycin and metronidazole. The results further verified the antimicrobial efficacy of argyrin B against toxigenic *C. difficile*, with MIC_90_ in the low ng/mL value of 25 ng/mL (range between 25 and 50 ng/mL) (Tables 1B and S3).

**Table 1:** (A) Minimal inhibitory concentrations (MICs) of argyrins B, C and D against *C. difficile* strains DSM 28645 and DSM 27147. Values depicted represent the mean and range of three independent experiments; (B) Minimal inhibitory concentrations (MICs) of argyrin B against *C. difficile* strains of various ribotypes. Values depicted represent the mean of two independent experiments (*n* = 51)

A				
	MIC (ng/mL) against *C. difficile*			
	DSM 28645		DSM 27147	
Compound	Mean	Range	Mean	Range
Argyrin B	18.75	12.5–25	37.5	25–50
Argyrin C	25	25	25	25
Argyrin D	75	50–100	75	50–100

B			
	MIC (ng/mL)		
	MIC_50_	MIC_90_	Range
Argyrin B	12.5	25	12.5–50

The disruption of gut microbiota caused by antibiotic treatment significantly contributes to *C. difficile* infections and complicates their management. Therefore, we investigated the effects of argyrin B on anaerobic bacterial species commonly found in the human gut, including *Bacteroides fragilis*, *Bifidobacterium bifidum*, *Clostridium butyricum*, *Clostridium scindens*, *Clostridium sporogenes* and *Clostridium perfringens* (Table S4). Notably, argyrin B did not inhibit the growth of any of the tested strains at concentrations up to 1 µg/mL (40-fold the MIC_90_). Taken together, these findings indicate a narrow spectrum of activity of argyrins against *C. difficile*, while other anaerobic members of the human gut microflora, including the clinically important human pathogen *C. perfringens*, appear unaffected.

### Frequency of Resistance (FoR) determination and whole genome sequencing analysis (WGS)

To investigate the potential for *C. difficile* to develop resistance to argyrins and the rate at which it might occur, we assessed the in vitro frequency of resistance (FoR) of *C. difficile* strain DSM 28645 when cultured in the presence of 100 ng/mL of argyrin B (4-fold MIC). *C. difficile* mutants with reduced susceptibility were selected at a frequency as low as 4.6 × 10⁻⁹ on blood agar containing 100 ng/mL argyrin B, calculated from four independent experiments (Table S5). The determined FoR of *C. difficile* against argyrin B is consistent with previously reported data for this compound and the Gram-negative pathogen *B. multivorans*, and approximately one log_10_ lower than that determined for *P. aeruginosa*^28^. The MIC values of the argyrin B-resistant variants harbouring *fusA2* mutations were determined (Table S6); all mutants exhibited MIC values > 8 µg/mL, confirming high-level resistance. Whole genome sequencing (WGS) of seven argyrin-resistant mutants identified a single nucleotide polymorphism (SNP) in the *fusA2* gene, resulting in an amino acid substitution in EF-G, thereby confirming EF-G as the target of argyrins in *C. difficile*, consistent with previous findings in *P. aeruginosa*. The specific substitutions identified in EF-G were: S407Y (mutants ARM22, ARM33, ARM44, and ARM55), P476Q (mutants ARM4 and ARM77), and A664V (mutant ARM11). Additional SNPs, complex mutations, and insertions were also detected and are reported in Table 2. Notably, mutations in *mgtA2* were also present in all mutants, suggesting a potential compensatory adaptation to argyrin exposure; however, the functional significance of these mutations remains unclear.

**Table 2:** Whole genome sequencing analysis (WGS) of seven argyrin B-resistant *C. difficile* mutants in comparison to CD630

Mutation type^a^	Gene name	Product name	Nucleotide change	Protein change	Mutant code
SNP	*fusA2*	Elongation factor G	C1220A	S407Y	ARM22, ARM33, ARM44 and ARM55
SNP			C1427A	P476Q	ARM4 and ARM77
SNP			C1991T	A664V	ARM11
SNP	NA	Intergenic region	NA	NA	ARM33 and ARM44
Complex					ARM44
Complex					ARM44
Complex					ARM44
Insertion					ARM44
Complex					ARM33 and ARM44
Complex					ARM33 and ARM44
SNP					ARM33 and ARM44
Complex					ARM33 and ARM44
Complex					ARM44
Deletion					ARM44
Complex					ARM44
Complex					ARM44
SNP					ARM44
SNP	NA	Intergenic region	NA	NA	ARM33, ARM44 and ARM55
Insertion	*CDIF630erm_03048*	Putative membrane protein	164–169 GCAATA duplication	S55-N56 duplication	ARM55
SNP	*CDIF630erm_03372*	PTS system, glucose-like IIBC component	A123T	K41N	ARM33, ARM44 and ARM55
SNP	*mgtA2*	Magnesium-transporting ATPase, P-type	T2204G	V735G	All seven mutants

*NA* not applicable, *SNP* single-nucleotide polymorphism.

^a^Complex mutations consist of several mutations in intergenic regions, where a functional impact cannot be assessed.

### Growth kinetics and toxin A + B production of argyrin B-resistant *C. difficile* mutants

**Fig. 1:**
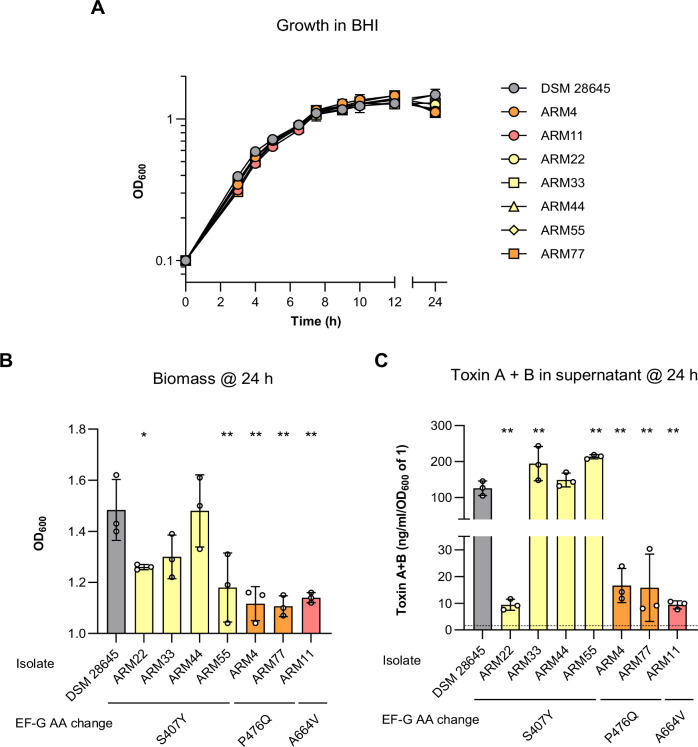
Impact of *fusA2* mutations on the fitness of *C. difficile* strain DSM 28645. **A** Growth kinetics of strain DSM 28645 and its isogenic *fusA2* mutants in BHI under static conditions. **B** Biomass formed after 24 h of growth in BHI. **C** Toxin A + B concentrations in supernatants of BHI cultures at 24 h of growth (normalized to the OD_600_ readings of the respective cultures at the sampling time point). Data are representative of the mean ± SD of three biological replicates. Dashed horizontal lines indicate the limit of detection. *P < 0.05; **P < 0.01 (one-way ANOVA with Dunnett’s multiple comparison test. Comparisons of the mean of the parental strain with the mean of the mutants are shown).

To assess whether argyrin-B resistance formation in *C. difficile* might be associated with a fitness cost, we determined the growth kinetics and toxin A + B production of strain DSM 28645 and the argyrin B-resistant mutants (Fig. 1). Culturing of the parental strain and its argyrin B-resistant mutants in BHI revealed no clear differences in the growth kinetics for the first 12 h of growth (Fig. 1A). However, at 24 h of growth, cultures of strain DSM 28645 produced significantly higher OD_600_ readings than most of the argyrin B-resistant mutants, except for mutant ARM44 (Fig. 1B), suggesting the parental strain to be able to produce a higher biomass than the tested argyrin B-resistant mutants under these growth conditions. Notably, significant differences between strain DSM 28645 and its argyrin B-resistant mutants were also observed for the toxin A + B contents found in the supernatants of the BHI cultures after 24 h of growth (Fig. 1C). The EF-G P476Q mutants ARM4 and ARM77 and the A664V mutant ARM11 produced only about 1/3 of the toxin A + B levels than the parental strain, suggesting that both *fusA2* mutations might be associated with some fitness cost. However, for the argyrin-resistant mutants ARM22, ARM33, ARM44 and ARM55, which all produce an EF-G variant with a serine to tyrosine exchange at amino acid position 407, a less clear picture emerged. While mutant ARM44 produced a toxin A + B content comparable to that of the parental strain, this was not the case for the other EF-G S407Y mutants tested here, which accumulated either lower (ARM22) or higher (ARM33, ARM55) toxin A + B contents in their supernatants after 24 h of growth in BHI.

### Evaluation of argyrin B pharmacokinetics in mice

**Fig. 2:**
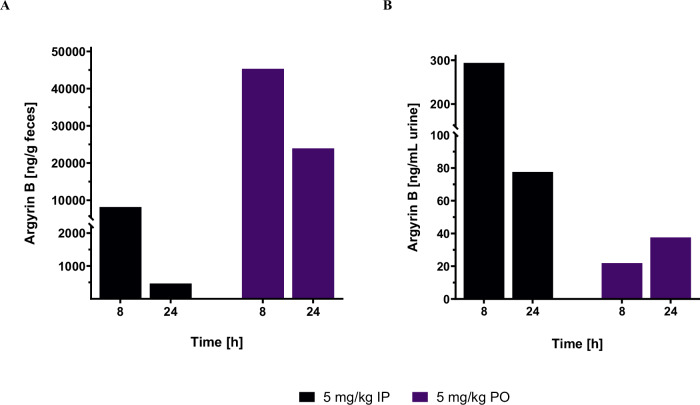
Pharmacokinetics of argyrin B in C57BL/6JRj mice. Concentration of argyrin B in faeces (**A**) and urine (**B**) at 8 and 24 h following *per os* (p.o.) and intraperitoneal (i.p.) administration.

Intrigued by the selective activity of argyrins, we evaluated the pharmacokinetic (PK) properties of argyrin B in mice to conclude on the therapeutic potential of the compound in treating CDI, which typically relies on oral antibiotic dosage. For this, male C57BL/6JRj mice were treated with a single *per os* (p.o.) dose of 5 mg/kg argyrin B and blood PK, urine and faeces levels were compared to those obtained from mice receiving a single intraperitoneal (i.p.) dose of 5 mg/kg (Figs. 2 and S2). Due to the physicochemical properties of argyrin B, we were expecting low oral bioavailability and indeed, blood levels were generally very low in the p.o. group as assessed between 0.25 h and 24 h post-dose, with concentrations in the range of 0.4–17 ng/mL. In contrast, after i.p. administration, we found higher initial exposure in blood (maximum plasma concentration (*C*_max_) = 915 ng/mL at 0.25 h post-dose) but rather fast clearance characteristics (area under the concentration–time curve (AUC) = 1470 ng h/mL, apparent clearance (CL/F) = 3420 mL/h/kg, elimination half-life (*t*½) = 3.4 h). The parent compound was detected at higher concentrations in faeces compared to urine (8.2 ng/g in pooled faeces 8 h post-dose) and some argyrin B was excreted renally (294 ng/g in pooled urine 8 h post-dose). Encouragingly, we found high levels in faeces after p.o. administration of argyrin B (45.3 ng/g at 8 h post-dose) and only very low amounts in urine (22 ng/mL at 8 h post-dose), in line with the compound’s poor resorption characteristics. Furthermore, argyrin B was still found in significant amounts 24 h after p.o. administration in faeces (23,936 ng/g), suggesting favourable retention in the gut after a single oral dose.

### In vivo activity testing of argyrin B in a *C. difficile* mouse model

**Fig. 3:**
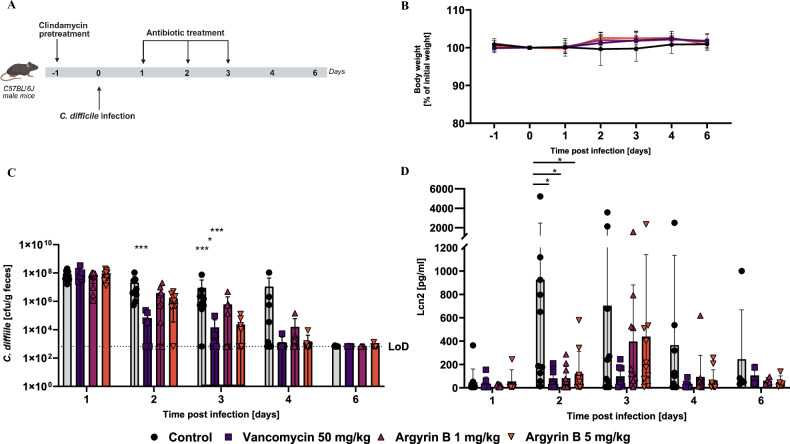
Argyrin B exerts activity against *C. difficile* in vivo. **A** C57BL/6J mice were treated intraperitoneally (i.p.) with clindamycin one day prior to infection with 2000 spores of *C. difficile* strain 630 by oral gavage. Mice were treated with argyrin **B**, vancomycin, or vehicle control on days 1, 2 and 3 post-infection by oral gavage. **B** Body weight was normalized to the time point of infection (day 0). **C** Faecal pellets were collected, homogenized and serial dilutions were plated on selective agar plates to quantify *C. difficile* titre. **D** Lipocalin-2 levels in faecal pellets were quantified by ELISA. **B**–**D** *n* = 7 –10 per group. Data are shown as mean ± SD. For **C** and **D**, data were log-transformed and analysed using a REML mixed-effects model with Dunnett’s multiple-comparison test. *P ≤ 0.05, ***P ≤ 0.001. LoD limit of detection. Only statistically significant differences are indicated.

In order to evaluate the in vivo efficacy of argyrin B against *C. difficile* infection, we used the clindamycin pretreatment model^42^. C57BL/6J mice were treated with a single dose of clindamycin intraperitoneally (i.p.). The following day, mice were infected with 2 × 10^3^ spores of *C. difficile* 630 (Fig. 3A). Colonization levels were evaluated by inoculating homogenized faecal pellets onto CLO agar plates with reduced oxygen levels. Mice were treated with two different doses of argyrin B (1 and 5 mg/kg) by oral gavage on days 1, 2 and 3 after infection. Control mice were treated with vancomycin (50 mg/kg), which is effective in killing *C. difficile* but also broadly affects commensal bacteria. Body weight of mice was not affected by either treatment (Fig. 3B). Argyrin B significantly reduced *C. difficile* burden in the faeces as compared to vehicle control mice on day 3 post-infection and was similar to the activity of vancomycin (Fig. 3C). The anti-*C. difficile* activity of argyrin B was also apparent on day 4 post-infection and was similar to the activity of vancomycin. In order to analyse *C. difficile*-induced intestinal inflammation, we measured Lipocalin-2 (Lcn-2)—a sensitive marker for intestinal inflammation—in faecal homogenates (Fig. 3D). Only few mice had elevated Lcn-2 levels on day one post-infection. In most mice, inflammation became apparent on day two post-infection. At this time point, *C. difficile*-infected, vehicle-treated control mice had high levels of Lcn-2, which were significantly lower in mice that were treated with vancomycin or either dose of argyrin B. From days 3 to 6, Lcn-2 levels declined, but there were no significant differences between treated and vehicle-treated mice.

### Faecal microbiota analysis following argyrin B treatment in mice

**Fig. 4:**
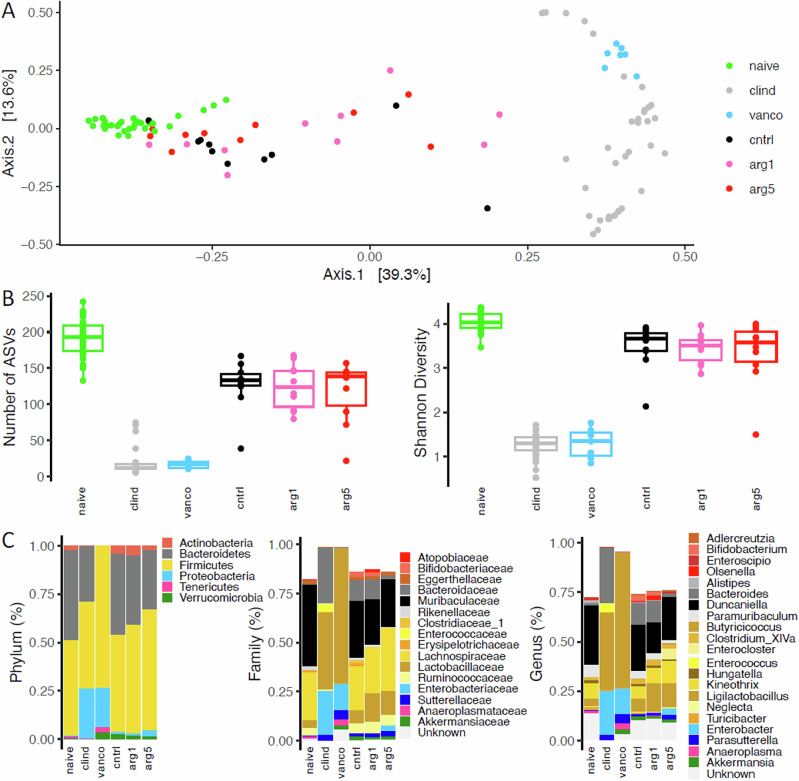
Microbiota analyses of mouse faecal samples before and after antibiotic treatment. Samples of naïve mice before any treatment (naïve), after clindamycin treatment (clind) and 4 days post-infection, where mice were either treated with vancomycin (vanco), argyrin B 1 mg/kg (arg1) or 5 mg/kg (arg5), as well as vehicle only (cntrl) were analysed. **A** Principal coordinate analysis based on Bray–Curtis dissimilarities. **B** Results of diversity indices (Richness and Shannon). **C** Average taxonomic compositions of individual groups at the phylum, family and genus level (only most abundant taxa are shown). ASV amplicon sequence variant.

To evaluate the specificity of argyrin B, we analysed its effect on the faecal microbiota. Faecal samples were collected from all mice at three time points: prior to clindamycin treatment (naïve group), one day after clindamycin treatment but before infection (clind group), and one day after the final treatment on day 4 post-infection with either vancomycin (vanco group), argyrin B (arg groups), or vehicle (Ctrl group). As expected, clindamycin-treated mice showed all highly dysbiotic microbiota compositions along with reduced diversities (Fig. 4A, B). Microbiota in mice treated with argyrin B displayed a similar pattern as control communities (vehicle treatment only), with compositions and diversities four days post-infection approaching those of baseline communities of naïve mice (before clindamycin treatment). In contrast, vancomycin-treated mice stayed highly dysbiotic throughout the experiment. Clindamycin- and vancomycin-treated mice were both enriched in *Proteobacteria*, mainly due to the genera *Enterobacter* and *Sutterella*, and lacked *Actinobacteria* (e.g., *Bifidobacteria*) (Fig. 4C). In the latter group, *Bacteroidetes*, mainly represented by the genus *Bacteroides*, were greatly reduced as well. Additionally, *Enterococcus* was detected at higher levels in clindamycin-treated mice, whereas *Akkermansia* of the *Verrucomicrobia* was lacking in those communities. Collectively, these data support the narrow-spectrum activity of argyrin B against *C. difficile* as restoration of communities was hardly affected by the substance at any concentration tested compared with the control group.

## Discussion

In this study, we demonstrate that argyrin B exhibits potent activity against *C. difficile*, both in vitro and in vivo, with efficacy comparable to vancomycin at substantially lower doses. Notably, argyrin B reduced faecal *C. difficile* burden after a single dose and preserved gut microbiota diversity to a greater extent than vancomycin. These findings establish argyrin B as a promising narrow-spectrum candidate for the treatment of CDI. Argyrins target EF-G, a highly conserved component of the bacterial translation machinery. The pronounced selectivity of argyrin B towards *C. difficile*, despite the conservation of EF-G across bacterial species, is therefore particularly intriguing. A similar phenomenon has been described for fidaxomicin, which inhibits the conserved RNA polymerase yet exhibits narrow-spectrum activity against *C. difficile*, attributed to a sensitizing residue absent in many gut commensals^43^. This precedent suggests that subtle structural or conformational differences in EF-G may contribute to the species-specific susceptibility observed here. Differences in EF-G isoforms, *fusA* paralog expression, membrane permeability, or efflux capacity may further influence susceptibility. Further structural and biochemical studies are required to define the molecular basis of this selectivity.

The narrow-spectrum activity observed in vitro was reflected in vivo, where argyrin B-treated mice retained key commensal taxa that were depleted following vancomycin treatment. Preservation of commensal anaerobes such as *Bacteroides*, *Bifidobacterium*, and *Duncaniella* may contribute to the maintenance of colonization resistance, potentially reducing recurrence risk. Whether this microbiota-sparing effect translates into reduced relapse in recurrent CDI models remains to be determined.

Resistance analysis revealed that argyrin B-resistant mutants arose at a low frequency and harboured point mutations in *fusA2*, confirming EF-G as the molecular target in *C. difficile*. Although resistance emerged via target modification, the observed frequency was lower than that reported for certain Gram-negative pathogens, suggesting a comparatively favourable resistance profile in *C. difficile*. Nonetheless, the clinical relevance of these mutations and their impact on fitness and virulence remain to be determined. Pharmacokinetic analysis demonstrated minimal systemic exposure and strong colonic accumulation, supporting the suitability of argyrin B for localized intestinal therapy. The effective dose required to achieve therapeutic benefit was markedly lower than that of vancomycin, highlighting its in vivo activity.

In our mouse model, spontaneous clearance of *C. difficile* was observed in untreated controls, indicating that this model does not fully recapitulate the persistence or recurrence patterns characteristic of human CDI. Alternative murine models employing more virulent strains and/or pretreatment with an antibiotic cocktail can induce more sustained colonization, greater weight loss, and more severe disease; however, these models are often associated with increased mortality. The respective advantages and limitations of available CDI models have been comprehensively reviewed by Brosse et al.^44^. More stringent relapse models, including hamster or minipig models, may provide additional insight into therapeutic efficacy, recurrence prevention, and translational potential.

Collectively, these findings highlight argyrin B as a promising EF-G-targeting antibiotic candidate with narrow-spectrum activity against *C. difficile*, favourable pharmacokinetics, and microbiota-sparing properties. Further studies are warranted to define its therapeutic window and efficacy in relapse models.

## Methods

### Strains and culture conditions

The bacterial strains *Bacteroides fragilis* DSM 2151, *Bifidobacterium bifidum* DSM 20456, *Clostridioides difficile* DSM 27147, CD630 DSM27543, and DSM 28645 (aka. CD630 *Δerm*), *Clostridium butyricum* DSM 10702, *Clostridium perfringens* DSM 796, *Clostridium scindens* DSM 5676, and *Clostridium sporogenes* DSM 795 were purchased from the German Collection of Microorganisms and Cell Cultures GmbH (DSMZ), Braunschweig, Germany. Clinical *C. difficile* isolates used in this study were obtained from the stock collection of the German National Reference Center for *Clostridioides difficile*, Homburg, Germany. Strains were cultured on Brucella blood agar with hemin and vitamin K1 (BBA; Becton Dickinson GmbH, Heidelberg, Germany) or in brain heart infusion broth (BHI, COPAN Diagnostics Inc., California, USA) in a Whitley A35 anaerobic workstation (Don Whitley Scientific Limited, West Yorkshire, UK) at 37 °C and a gas mixture of N_2_ (90.9%), CO_2_ (9.0%) and O_2_ (0.1%).

### Antimicrobial susceptibility testing

For library screening against *C. difficile* in 96-well plates, a microdilution assay was performed with modified standard inoculum and prolonged incubation time as compared to CSLI^45^. Suspension of isolates was adjusted to McFarland 1 and diluted 1:100 in BHI for testing. For every experiment, positive growth controls were mandatory to prove the validity of the initial screening test. All compounds were screened at 10 µM final concentration in four technical replicates. Hits were defined as compounds that prevented visible growth of *C. difficile* after 3 weeks of incubation. The minimal inhibitory concentrations (MICs) of argyrin derivatives tested here were determined by the broth microdilution method following the Clinical and Laboratory Standards Institute (CLSI) protocol M11-A8 (CLSI 2012)^45^, however, with BHI instead of Brucella broth supplemented with hemin, vitamin K1, and lysed horse blood. Test substances were dissolved in dimethyl sulfoxide (DMSO; Carl Roth, Karlsruhe, Germany) or a polyethylene glycol 400 (PEG 400; Merck KGaA, Darmstadt, Germany)/phosphate-buffered saline (PBS; Gibco, Thermo Fisher, Germany) mixture as indicated. Final concentrations of DMSO and PEG400 in the assay were ≤1%.

### Frequency of resistance (FoR) determination

The selection of mutants with decreased susceptibility to argyrin B was carried out with *C. difficile* strain DSM 28645. Cells of this strain were inoculated into 10 mL of fresh BHI and the culture was grown until its turbidity reached a McFarland of 4. Cells were harvested by centrifugation at 5000 rpm for 10 min, the cell pellet was washed once with PBS, and cells were resuspended in 0.5 mL of PBS to reach a cell density of about 5E + 09 to 1E + 10 CFU/mL. 100 µL aliquots of the bacterial solution (~5E + 08 to 1E + 9 cells/plate) were plated on BBA plates supplemented with fourfold MIC of argyrin B (i.e., 100 ng/mL) and incubated anaerobically at 37 °C for 48 h. Another 100 µL aliquot of each cell suspension was serially diluted (tenfold) in PBS and plated out on drug-free BBA plates for CFU enumeration. All colonies grown on the argyrin B-containing BBA plates were transferred to a fresh argyrin B-containing BBA plate (100 ng/mL) to confirm the ability of the mutant to grow in the presence of elevated argyrin B concentrations (4× MIC). A colony of the wild-type strain served as an argyrin B activity control. The FoR was calculated as the number of argyrin B-resistant mutants found on the drug-containing plates divided by the number of CFUs that were plated onto the drug-free BBA plates^28^.

### Whole genome sequencing

For whole genome sequencing (WGS) and subsequent comparative genomics, pure cultures of the resistant mutants were subjected to DNA extraction using the Monarch Genomic DNA Purification Kit (New England Biolabs, Ipswich, MA, USA). Genomic DNA was sequenced on a PacBio Sequel IIe system using the SMRTbell Express Template Prep Kit 2.0 (Pacific Biosciences Inc., Menlo Park, CA, USA). Sequencing data were assembled de novo using the SMRT Link software suite 10 (Pacific Biosciences Inc.) with default parameters as recently described^46^. Mutations were determined in comparison to the genome sequence of CD630 Δ*erm* using the SeqSphere^+^ software version 8 beta (Ridom GmbH, Münster, Germany).

### Growth kinetics

For the determination of the growth kinetics of *C. difficile* strain DSM 28645 and its isogenic argyrin B-resistant mutants, pre-cultures were prepared by suspending 3 colonies of the respective isolates (taken from freshly grown BBA plates) in 3 mL of BHI that was allowed to equilibrate to the anaerobic environment for at least 12 h. Pre-cultures were cultivated for 24 h under static conditions, and subsequently used to inoculate 30 mL of fresh BHI medium to an OD_600_ of 0.1. Main cultures were incubated in 50 mL Falcon-tubes at 37 °C for 24 h, and aliquots were removed after 3, 4, 5, 7.5, 9, 12 and 24 h of growth under static conditions for OD_600_ determinations.

### Toxin A + B determination

The amounts of toxin A (TcdA) and B (TcdB) in culture supernatants of *C. difficile* BHI cultures were measured by an enzyme-linked immunosorbent assay using the tgcBIOMICS kit TGC-E001-1 (Antibodies-online, Aachen, Germany) for the simultaneous detection of toxin A + B. Briefly, *C. difficile* isolates were grown in BHI as outlined above. After 24 h of growth, aliquots of the cultures were centrifuged, the supernatants collected and stored at −70 °C until further usage. On the measurement days, supernatants were thawed on ice, diluted in the dilution buffer provided by the kit and assayed according to the manufacturer’s instructions. Toxin levels were determined in reference to a standard curve obtained with the toxin A + B standard provided by the kit and normalized to the OD_600_ readings of the respective cultures at the sampling time point.

### Non-GLP Ethics statement

Mice were maintained under specific-pathogen-free conditions in individually ventilated cages, with standard chow and water provided ad libitum. Pharmacokinetic studies were conducted at Pharmacelsus GmbH (Saarbrücken, Germany) in accordance with the regulations of the local animal welfare authority (Landesamt für Gesundheit und Verbraucherschutz, Abteilung Lebensmittel- und Veterinärwesen; approval TV 2.4.2.2 06/2023). Mouse infection experiments were performed in the animal facilities of Hannover Medical School (Germany) in full compliance with the German Animal Protection Law and with approval from the Animal Care Committee of the Niedersächsisches Landesamt für Verbraucherschutz und Lebensmittelsicherheit (protocol no. 21/3702).

### Pharmacokinetic evaluation of argyrin after following *per os* (PO) or intraperitoneal (IP) administration to male C57BL/6 J mice

Adult male C57BL/6J mice (7 weeks old; Janvier Labs, France) were group-housed under controlled conditions (20–24 °C, 12 h light/dark cycle) with food and water ad libitum. Argyrin B was freshly prepared and administered orally (5 or 10 mg/kg) or intraperitoneally (1 or 5 mg/kg). Blood samples (100 µL) were collected from three animals per time point (15, 30 min, 1, 3, 6, 8, and 24 h) via retro-orbital puncture under isoflurane anaesthesia into heparinized tubes. Plasma was obtained by centrifugation (10 min, 4 °C, 4500×*g*), stabilized with methanolic PMSF (5 mg/mL; 1:10 v/v), and stored at −20 °C until LC–MS analysis. Faeces and urine were collected at 8 and 24 h (*n* = 3 per condition). LC-MS analysis was performed using a Dionex UltiMate 3000 RS HPLC coupled to a Q Exactive Plus Orbitrap mass spectrometer with heated electrospray ionization (H-ESI) operated in positive mode. Data acquisition was conducted in full scan and parallel reaction monitoring (PRM, targeted MS/MS) mode. Separation was achieved on an Accucore PFP column (50 × 2.1 mm, 2.6 µm) with a C6-Phenyl precolumn using a gradient of acetonitrile/water containing 0.2% HFBA at 600 µL/min. Argyrin B eluted at 1.47 min. Quantification was performed using PRM with an isolation window of 40 amu. The precursor ions monitored were [M + H] + *m*/*z* 839.3302 and [M+Na] + *m*/*z* 861.3310. Collision energy was 10 eV, dwell time 100 ms, maximum injection time 80 ms, and mass tolerance 10 mmu. The instrument was internally calibrated using diisooctyl phthalate ([M + H] + *m*/*z* 391.28429) as a lock mass. Source parameters were: spray voltage 4 kV, capillary temperature 350 °C, sheath gas 45 psi, auxiliary gas 20 psi, and sweep gas 2 psi. Column temperature was maintained at 30 ± 1 °C.

### Mouse infections

C57BL/6 J mice were bred in the animal facility of Hannover Medical School. *C. difficile* strain 630 spores were prepared according to Edwards et al.^47^ and stored at 4 °C. Argyrin B was dissolved in DMSO and diluted gradually with warm 0.5% methylcellulose solution in an ultrasonic bath at 37 °C; the final concentration of DMSO was 5%. One day before infection, male C57BL/6 J mice (14–16 weeks old) were injected intraperitoneally with 250 µg clindamycin in 100 µL PBS. 24 h later, mice were infected with 2 × 10^3^ *C. difficile* 630 (DSM 27543) spores in a volume of 100 µL by oral gavage. One, two and three days after infection, mice were treated with argyrin B (1 or 5 mg/kg body weight) or vancomycin (50 mg/kg dissolved in H_2_O) in a volume of 100 µL by oral gavage. The control group received 100 µL of vehicle (0.5% methylcellulose, 5% DMSO). Weight and health conditions of mice were monitored and scored daily. Fresh faecal pellets were collected in 500 µL of oxygen-reduced PBS at different time points and the weight was measured. A 5 mm stainless steel bead (Qiagen) was added and faecal pellets were homogenized for 5 min at 30 Hz using a TissueLyser II (Qiagen). Serial dilutions of the homogenate were plated on oxygen-reduced CLO plates (Biomerieux 43431). The plates were incubated for 1–2 days under anaerobic conditions (10% CO_2_, 10% H_2_, 80% N_2_). *C. difficile* colonies were counted, calculated as colony-forming units (CFU) per gram faeces, logarithmically transformed and analysed using restricted maximum likelihood mixed effects model with Geisser–Greenhouse correction and Dunnett’s post-test (Graphpad Prism V8.0).

### Lipocalin-2 ELISA

The remaining faeces homogenate was centrifuged for 2 min at 13,000 rpm and 4 °C to pellet solid components and the supernatant was frozen at −20 °C. Mouse Lipocalin-2/NGAL DuoSet ELISA (R&D Systems) was performed with the supernatant according to the manufacturer’s protocol. Data were logarithmically transformed and analysed using one-way ANOVA with Dunnett’s post-test.

### Microbiota analysis

Processing of samples was done according to Kircher et al.^48^. In brief, DNA extraction of faecal pellets was achieved with the ZymoBIOMICS DNA Kit (Zymo Research, Irvine, CA, USA), followed by a two-step PCR amplification targeting the V3V4 region of the 16S rRNA gene and subsequent sequencing on Illumina MiSeq in paired-end mode (2 × 250 bp). Filtering, merging of reads, chimera removal and amplicon sequence variant classification based on RDP were done in DADA2 ^49^ as described previously (PMID: 36416760). Calculations of diversity indices (Richness and Shannon) and principal coordinate analyses, based on Bray–Curtis dissimilarities, were performed in R using the package phyloseq. Analyses are based on proportional count data, except for Richness estimations, where samples were rarified to equal depth (6856 sequences per sample) before analysis.

## Supplementary information

Supplementary Information

## Data Availability

The data supporting the findings of this study are available within the article and its Supplementary Information files.
