# Supplementary material for: Argyrin B exhibits potent therapeutic efficacy in a Clostridioides difficile-infection mouse model while preserving microbiota functionality
Source: NPJ Antimicrob Resist. 2026 Jul 16;4:44. doi: 10.1038/s44259-026-00248-x (PMC13392398; doi:10.1038/s44259-026-00248-x)
Supplement: Supplementary file 1 — Supplementary Information [file 44259_2026_248_MOESM1_ESM.pdf]

Supporting Information

Argyrin B Exhibits Potent Therapeutic Efficacy in a *Clostridioides difficile*-Infection Mouse Model while Preserving Microbiota Functionality

Sari Rasheed[1,2,3], Katrin Ehrhardt[2,4], Ahmed Mostafa Abdrabou[5,6,7], Alexander Mellmann[7,8], Anna Lechleiter[5], Domen Pogorevc[1,2], Sabryna Junker[1,2,5], Lutz von Müller[5,7,9], Marius Vital[2,4,11], Jennifer Herrmann*[1,2,3], Guntram A. Grassl*[2,4], Markus Bischoff*[1,2,5,7], Rolf Müller*[1,2,3,10,12]

1 Department of Microbial Natural Products, Helmholtz Institute for Pharmaceutical Research Saarland (HIPS)/Helmholtz Centre for Infection Research (HZI), Saarbrücken, Germany
2 German Center for Infection Research (DZIF), partnersite Hannover-Braunschweig, Germany
3 PharmaScienceHub (PSH), Saarland University Campus, Saarbrücken, Germany
4 Institute of Medical Microbiology and Hospital Epidemiology, Hannover Medical School, Hannover, Germany
5 Institute for Medical Microbiology and Hygiene, Saarland University, Homburg/Saar, Germany
6 Medical Microbiology and Immunology Department, Faculty of Medicine, Mansoura University, Mansoura, Egypt
7 German National Reference Center for *Clostridioides (Clostridium) difficile*, Homburg/Saar–Münster–Coesfeld, Germany
8 Institute of Hygiene, University of Münster, Münster, Germany
9 Institute for Laboratory Medicine, Microbiology and Hygiene, Christophorus Kliniken, Coesfeld, Germany
10 Department of Pharmacy, Saarland University, Saarbrücken, Germany
11 Cluster of Excellence RESIST (EXC 2155), Hannover Medical School, Hannover, Germany
12 Responsible corresponding author

Rolf Müller is the responsible corresponding author
Email: rolf.mueller@helmholtz-hips.de

*Corresponding authors:
Jennifer Herrmann: jennifer.herrmann@helmholtz-hips.de (J.H.),
Guntram Grassl: grassl.guntram@mh-hannover.de (G.A.G)
Markus Bischoff: markus.bischoff@uks.eu (M.B.)
Rolf Müller: rolf.mueller@helmholtz-hips.de (R.M.)

Keywords: argyrin B, *Clostridioides difficile*, elongation factor G, mouse infection model, microbiota preservation, gut-targeted therapy

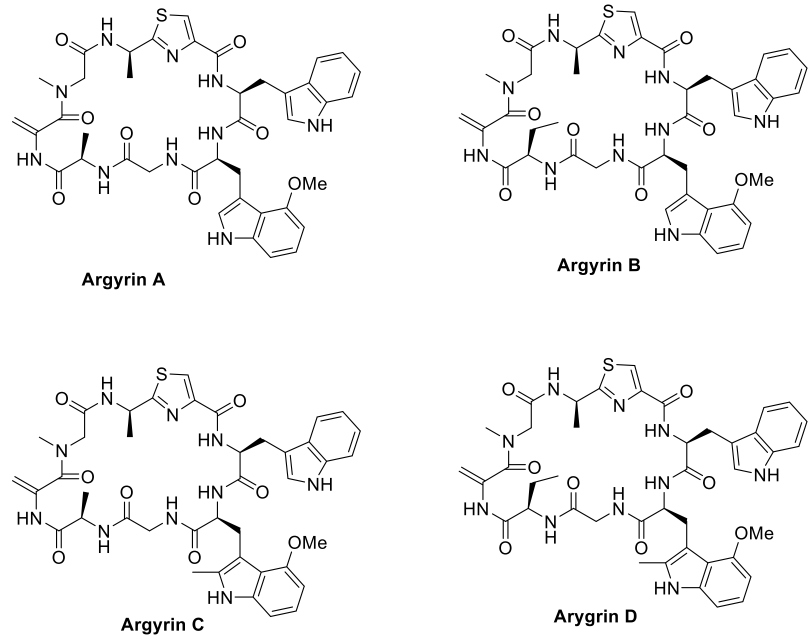

Figure S1. Chemical structures of argyrins A-D.

Table S1. Prioritization of primary hits from library screening of 259 natural products from myxobacteria. Listed compounds fully inhibited the growth of *C. difficile* DSM 27147 at 10 µM assay concentration (n=4). NA: not applicable.

**Compound name**	**Molecular weight [g/mol]**	**Prioritized**	**Deprioritization criterium**
Argyrin A	824.9	X	NA
Argyrin B	838.9	X	NA
Argyrin C	838.9	X	NA
Argyrin D	852.6	X	NA
Chlorotonil C	419.0		known activity against *C. difficile*
Corallopyronin A	527.6		prioritized development as antifilarial agent
Icumazol B2	715.8		insufficient availability
Katanosin B	1276.5	X	
Leupyrrin A2	736.9		insufficient availability
Pentacaronic acid	486.6		insufficient availability
Sorangicin A	807.0	X	

Neosorangicin A	751.0		
Soraphen D	494.6		insufficient availability
Tartrolon B	856.8		toxicity alert
Tartrolon C	910.8		toxicity alert
Terrestribisamid B	444.5		insufficient availability
Tybulysin Y	729.9		cytotoxic

Table S2. Dose-Response activity of selected compound classes against *C. difficile* DSM 27147 and B. fragilis DSM 2151. Compounds were tested (n=3) in serial dilution (0.03-64 µg/mL) to determine minimum inhibitory concentrations (MICs).

**Compound**	**MIC [µg/mL]**	
	***C. difficile* DSM 27147**	***B. fragilis DSM 2151***
Argyrin A	≤ 0.03	> 64
Argyrin B	≤ 0.03	> 64
Argyrin C	≤ 0.03	> 64
Argyrin D	≤ 0.03	> 64
Argyrin F	4	> 64
Argyrin H	0.25	> 64
Argyrin I	1	> 64
Sorangicin A	0.5	4
Katanosin B	0.25	8

Table S3. MIC values of 51 *C. difficile* strains of various ribotypes for argyrin B, vancomycin, and metronidazole. Data represent the mean of two independent experiments. **Strains tested for argyrin B dissolved in PEG 400/PBS displayed MIC values between 100-200 ng/mL. #Strains were PCR-negative for tcdA, tcdB and cdtAB. ND: not determined.*

**Strain code**	**Ribo-type**	**MIC [ng/mL]**	**MIC [µg/mL]**		**Resistance phenotype**		
		**Argyrin B**	**Vanco-mycin**	**Metro-nidazole**	**Moxi-floxacin**	**Clari-thromycin**	**Rif-ampicin**
A	RT027	25	0,5	0,75	R	R	S
B	RT027	25	0,75	0,75	R	R	R
C	RT027	12,5	1	1	R	R	R
D	RT027	25	1,5	1	R	R	S

E	RT027	25	0,75	1,5	R	R	S
F	RT027	12,5	0,75	1	R	R	R
G	RT027	25	0,5	2	R	R	S
H	RT027	12,5	2	1	R	R	R
I	RT027	12,5	1,5	0,75	R	R	R
J	RT027	12,5	0,5	1	R	R	S
K	RT078	25	0,38	0,38	S	R	S
L	RT078	12,5	0,5	0,38	R	S	S
M	RT078	12,5	0,38	0,125	R	R	S
N	RT078	12,5	0,38	0,75	R	R	S
O	RT078	18,75	0,38	0,75	R	S	S
P	RT014	12,5	1	0,5	R	S	S
Q*	RT014	12,5	1,5	1	R	R	S
R*	RT014	12,5	0,5	0,5	R	S	S
S	RT014	25	0,38	0,75	S	R	S
T*	RT014	12,5	0,25	0,75	S	R	S
V	RT001	12,5	1	1	R	R	S
W	RT001	18,75	1	0,5	S	S	S
X	RT001	12,5	0,38	0,5	R	R	S
Y	RT001	12,5	0,75	1,5	R	R	S
Z*	RT001	25	0,38	0,25	R	R	S
AA*	RT001	12,5	0,38	0,125	R	R	S
AB	RT001	25	1	0,5	R	R	S
AC*	RT001	12,5	0,25	0,5	R	R	S
AD	RT018	12,5	0,38	0,5	S	S	S
AE	RT018	12,5	0,38	0,5	R	S	S
AF	RT176	50	0,38	2	R	S	S
AG*	RT176	18,75	0,094	0,75	R	R	R
AH*	RT176	25	0,38	1,5	R	R	S
AI*	RT176	25	0,5	1	R	R	S
AJ*	RT176	25	0,5	1,5	R	R	S
AK*	RT106	12,5	0,75	1,5	R	S	S
AL	RT106	18,75	0,75	0,75	S	R	S
AM	RT106	12,5	0,75	1	S	S	S
AN	RT106	12,5	0,38	0,38	S	S	S
AO	RT017	37,5	0,25	0,38	R	S	R
AP	RT017	12,5	1,5	0,38	R	R	R
AQ[#]	RT010	12,5	0,25	0,125	S	R	S
AR[#]	RT140	18,75	0,38	1	R	R	S
AS*[#]	RT002	12,5	ND	ND	ND	ND	ND
AT	RT002	12,5	0,38	0,75	S	S	S
AV	RT002	25	0,5	0,75	R	S	S
AW	RT002	12,5	0,5	0,75	S	S	S
AX	RT020	18,75	0,5	0,25	S	S	S
AY	RT020	25	0,5	0,25	R	S	S
AZ*	RT020	12,5	0,75	1	S	S	S
AAA	RT020	12,5	0,5	0,75	S	S	S

**MIC_{50}**	**12.5**	**0.5**	**0.75**
**MIC_{90}**	**25**	**1**	**1.5**

Table S4. MIC values of Argyrin B against strains of common gut colonizing species. Data represent the mean of two independent experiments.

**Species**	**Strain**	**MIC [µg/mL]**
		**argyrin B**
Bacterioides fragilis	DSM 2151	>1
Bifidobacterium bifidum	DSM 20456	>1
Clostridium butryticum	DSM 10702	>1
Clostridium scindens	DSM 5676	>1
Clostridium sporogenes	DSM 795	>1
Clostridium perfringens	DSM 796	>1

Table S5. *In vitro* frequency of resistance of *C. difficile* strain DSM 28645 grown on tryptic soy blood agar in presence of 100 ng/ml argyrin B.

**Experiment**	**Number of colonies on selective plate**	***C. difficile* cells inoculated per plate**	**Mutant frequency**
**a**	1	5E+08	2.00E-09
**b**	2	6E+08	3.33E-09
**c**	1	5E+08	2.00E-09
**d**	8	1E+09	8.00E-09
**Total**	**12**	**2.6E+09**	**4.62E-09**

Table S6. MIC values of argyrin B-resistant mutants. Data represent the mean of two independent experiments.

**Mutant code**	***fusA2* mutation**		**MIC [µg/mL]**
	**Nucleotide change**	**Protein change**	**Argyrin B**
ARM4	C1427A	P476Q	>8
ARM11	C1991T	A664V	>8
ARM22	C1220A	P476Q	>8

ARM33	C1220A	P476Q	>8
ARM44	C1220A	P476Q	>8
ARM55	C1220A	P476Q	>8
ARM77	C1427A	P476Q	>8

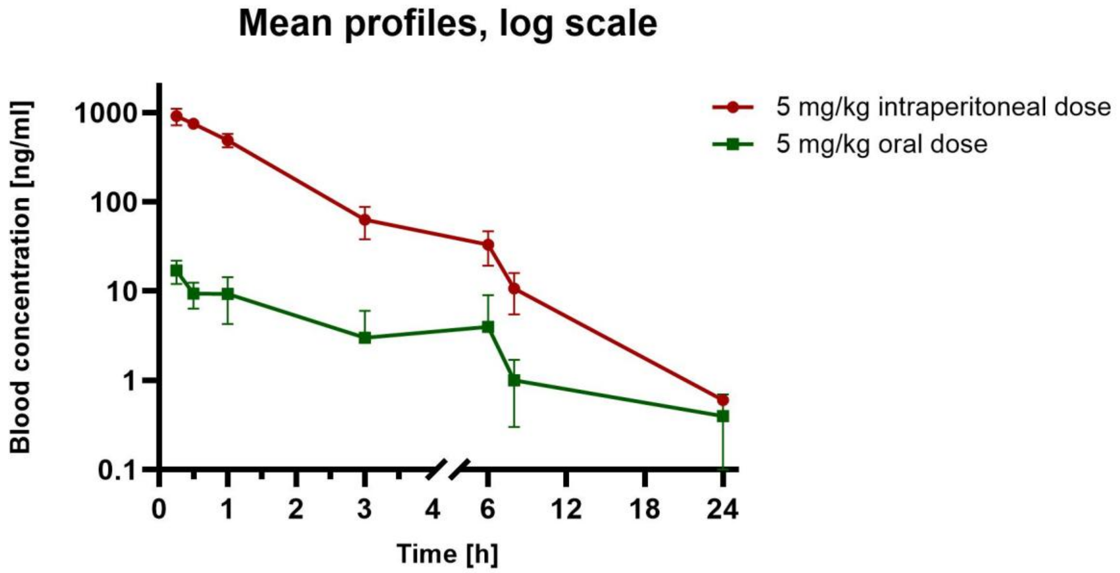

Figure S2. Blood concentration of Argyrin B in male C57BL/6J mice after intraperitoneal and oral administration of 5 mg/kg. Data presented on a logarithmic scale. Data represent the mean profiles of 3 mice.
